# Genito-Urinary Tuberculosis in the Female

**Published:** 1904-06

**Authors:** E. G. Jones

**Affiliations:** Atlanta, Ga.


ATLANTA

Journal-Record of Medicine.

Successor to Atlanta Medical and Surgical Journal, Established 1855,
and Southern Medical Record, Established 1870.

VOL. VI. JUNE, 1904. No. 3.

BERNARD WOLFF, M.D., EDITOR, Nos. 319–20 Prudential.

M. B. HUTCHINS, M.D., BUSINESS MANAGER, 1007–1008 Century Bldg.

Published Monthly.

### ORIGINAL COMMUNICATIONS.

### GENITO-URINARY TUBERCULOSIS IN THE FEMALE.

BY E. G. JONES, M.D.,
ATLANTA, GA.

### I. GENITAL TUBERCULOSIS.

It appears that up to 1831 genital tuberculosis was looked upon as a mere clinical curiosity. In fact, it may be fairly stated that the intelligent study of the subject dates from Koch's discovery of the tubercle bacillus. Almost immediately the bacillus was found in the vaginal secretions; and in 1886 Hegar furnished to the profession a contribution on genital tuberculosis which may be called a starting-point for all the immediately subsequent investigative and literary efforts upon the subject in question.

*Frequency.*—When one remembers the latency of the disease and the frequency with which women are permitted to die without a proper diagnosis of many less obscure maladies, a very natural, and even necessary, conclusion, though it lack proof, is that probably half the cases are overlooked.

Considering the ubiquitous character of tuberculosis it would seem that, excepting renal tuberculosis and testicular tuberculosis in the male, this disease in the genito-urinary tract has received but scant consideration at the hands of the profession, at least in this country.

Of all women dying of any disease 1 or 2 per cent. are said to have genital tuberculosis. The average of figures which I have collected from eight observers indicates that of all women dying of general tuberculosis, one in thirty have tuberculosis in some part of the genital tract, whether primary there or not. This is a rather low estimate. As to the frequency of tuberculosis, primary in the genital tract, there is the widest variation in figures given by different authorities. Three of these (Frerichs, Mosler, Spaeth), whom I quote through Murphy, give the figures as 6 per cent., 19.5 per cent. and 24.5 per cent. respectively.

Still observes that 9.5 per cent. of all tuberculous girls under twelve years are affected with genital tuberculosis. According to Amann (cited by Murphy) genital tuberculosis occurs seven times as often in the female as in the male.

The order of frequency with which the different parts are attacked is as follows: tubes, uterus, ovaries, vagina, vulva. Targett says the tubes are involved in 90 per cent. of cases of genital tuberculosis. In fact, he believes that 8 or 10 per cent. of all cases of salpingitis are tuberculous in origin. Since the tubes are affected so much more frequently than any other of the genital organs we must (confessing our ignorance) regard them as so-called "seats of election," and class them with the synovial membranes and epiphyses of bones as structures in which, for some unknown reason, tuberculosis is particularly liable to develop.

*Mode of Infection.*—It is, I believe, no longer doubted that tuberculosis may be primary in the genitalia. The statement is supported by at least two very convincing circumstances: (1) persons in otherwise perfect health have manifestations in the genital tract and nowhere else; (2) persons ill of genital tuberculosis are often entirely cured by ablation of the local focus.

As might be supposed, the secondary form is more common than the primary. One out of fourteen women suffering from genital tuberculosis seem to have it in its primary form; and it ought to be added that many observers deny absolutely the existence of any except the secondary type. This latter is not uncommon in phthisis.

The methods by which the bacilli reach the genitalia and there inaugurate the disease is a matter of much interest. They may reach the locality in question (1) by the blood or lymph from other parts of the body, (2) by continuity of surface, (3) by direct infection.

It needs no argument to prove hematogenous and lymphatic infection. The transmission of the disease from mother to fetus, the comparatively frequent involvement of the placental site, and tuberculous genital lesions sequent upon phthisis pulmonalis are occurrences the significance of which can scarcely be misinterpreted.

The involvement of a tube, or ovary, or even the uterus from an adjacent loop of intestine, or from the peritoneum similarly affected, is not uncommon.

Direct infection may take place from peritoneal fluid entering the fimbriated extremity. Probably more than 50 per cent. of women having tuberculous salpingitis have also tuberculous peritonitis (Targett). The extension is from peritoneum to tubes; not *vice versa*, else women would be much more subject to tuberculous peritonitis than men, which seems not to be the case.

Direct infection may also be caused by contaminated hands, or instruments, or clothing, or feces, or urine, and especially by contaminated semen. It is impossible for one to conclude, from the literature he may consult, whether or not the genital tract may be infected by the direct deposition of bacilli without antecedent trauma. It seems not, though experiments can be cited which apparently prove either side of the question. Popoff, from a series of experiments on guinea-pigs, concludes (1) that it is impossible to infect the genital organs unless there be some previous trauma; and (2) that in cases of tuberculosis following traumatism the lesions remain localized in the genital apparatus and neighboring lymph-glands. The latter conclusion seems not altogether warranted if we admit that many, if not a majority of, cases of genital tuberculosis result directly from the transmissibility of the disease by the blood.

It is undoubtedly a fact that women may be locally infected by semen from tuberculous testicles or seminal vesicles. It has been urged, furthermore, and with some plausibility, that women may be locally infected by the semen of tuberculous men not suffering from genital tuberculosis. Murphy quotes the experiments of Landouzy and Martin, who took semen from a tuberculous patient whose testicles were apparently healthy and injected it into guinea-pigs, with the result that one-third of the pigs so treated died of tuberculosis. Tubercle bacilli were also found in the testicular secretions of animals which had previously received injections of tuberculous cultures. However this may be, the fact remains that the majority of women having husbands with general or genital tuberculosis do not themselves contract genital tuberculosis.

*Diagnosis.*—In making a diagnosis practically the only sign worthy of dependence is the presence of bacilli. Every one knows that their absence at numerous examinations does not negative the diagnosis. It is hardly necessary to refer to the precautions necessary to differentiate between smegma and tubercle bacilli. If no germs are found after repeated trials, inoculation of a guinea-pig or rabbit with some of the suspected secretion is a satisfactory and not a difficult procedure. I need not mention the inquiry to be made into the constitutional symptoms of the patient, nor to the information to be obtained from that most valuable of all our clinical instruments, the thermometer. It is unquestionably often a matter of deciding if the genital be not merely a part of general tuberculosis.

I have seen somewhere an intimation that tubal pregnancy is suspiciously coincident with, if not often dependent upon, tubal tuberculosis.

*Symptoms.*—Sterility is mentioned as the most nearly invariable symptom. The menstrual function is not disturbed early if the genital involvement be primary; later amenorrhea and menorrhagia are said to occur with equal frequency. Pain is not a prominent symptom, nor is there any characteristic discharge. In fact, it may be stated that the usual insidious character of pulmonary tuberculosis is exaggerated when the seat of lesion is in the genitalia.

### II. URINARY TUBERCULOSIS.

Chronic urinary tuberculosis commonly originates in the kidney, involvement of the ureter and bladder being almost invariably secondary to renal infection. A most important fact is that the disease is usually monolateral if primary in the kidney. The opposite gland often, if not usually, becomes similarly involved unless the first be removed or the disease be checked in some other way. In such cases the bladder is involved as an intermediate agent. If the disease attack the bladder first of the urinary tract, both kidneys commonly become infected more or less simultaneously. It is to be remembered, however, that cystic origin is exceedingly rare. Hunner, reviewing thirty-five cases of urinary tuberculosis in women at the Johns Hopkins hospital, believes that not more than five—possibly only one—were primary in the bladder. E. J. Ill reports one out of his fourteen as being possibly primary in the bladder. When both kidney and bladder are involved it is impossible to say in which organ the disease began; but the known frequency of renal tuberculosis as compared with cystic, and the favorable course pursued by involved bladders after removal of the affected kidney, would certainly incline one, in case of doubt, to absolve the bladder from original sin.

Of the thirty-five cases referred to above, eleven are reported as having suffered from bladder tuberculosis in addition to the renal lesion. Seven others had some inflammation about the ureteral orifice, which inflammation subsided upon the removal of the affected kidney and was, therefore, non-tuberculous. Of fourteen cases of renal tuberculosis in women recently reported by Ill, there was involvement of bladder and ureter in nine; one other had bladder but no ureteral trouble. Ill calls all these bladders and ureters tuberculous. It ought to be stated here that the average time during which these patients had been suffering symptoms was about three and one-half years.

From the cases which I have been able to collect, the disease seems to be slightly more common on the right than on the left, though the Johns Hopkins series shows a majority of one for the left side. It is not stated how many of these women had borne children. Furthermore, the literature would seem to indicate that urinary tuberculosis is more common in white than in colored women, but this I think we will all doubt.

*Symptoms.*—In a discussion of the symptoms we must consider pain, bladder disturbances, urinary findings, physical signs, and the general condition of the patient.

In eight out of nine cases reported in the *Annals of Surgery* for October (Ill) the most prominent early symptom was pain in the lumbar region. Almost all suffered later from dysuria and hematuria. In Hunner's thirty-five cases the initial symptoms were referred to the bladder in seventeen; eleven complained first of lumbar pain, and five were first attacked with renal colic. The last-named mode of onset is reasonably explained by the attempted passage of caseous plugs through thickened, tender ureters.

Bladder symptoms are almost invariable in some stage of the disease. There may be no other. They vary from slight burning at the end of micturition to the most agonizing strangury. These vesical symptoms are not necessarily to be interpreted as indicating an actual bladder lesion.

Hematuria and pyuria may also be looked upon as invariable accompaniments. The blood and pus may come from the bladder or kidney, or both.

In about one-third of the cases the kidney is distinctly enlarged at the time the patient seeks advice; in a certain additional number the organ can be palpated. Such palpation often causes pain and a desire to urinate. A tuberculous ureter is tender and can commonly be felt as it passes through the pelvic brim; it can almost always be felt through the vagina or rectum.

The general condition of the patient may remain good for a long time, but she eventually exhibits systemic symptoms common to all forms of tuberculosis, colored of course by disturbances necessarily incident to progressive inroads upon the functional activity of the gland.

The discovery of tubercle bacilli in urine is sometimes a tedious procedure. Dilution of the urinary sediment with distilled water is said to aid materially in this discovery. But when they have been found a conclusion must be reached as to their source. They may come from the bladder, or from one kidney, or from both. It may, in most cases, be very safely presupposed that they have a renal origin. And I think I may safely state that it can usually be determined, with a fair degree of clearness, from which side they come without resort to ureteral catheterization. However, removal of the wrong kidney is so costly an error, that I think the average physician would hardly feel justified in failing to verify his diagnosis by resort to this procedure. Cystoscopic examination will almost always make one more suspicious of one kidney than the other. Indeed he is, well-nigh always, already reasonably confident as to which kidney is affected. The ureteral orifice corresponding to the diseased gland and the adjacent mucous membrane will often be a little inflamed and puffy, if not actually ulcerated. If the ureter happen to be also affected the site of its orifice may be a little depressed. This is the ureter into which the catheter is to be passed; the other should not be molested. If, now, after the catheter has been inserted, the bladder be thoroughly irrigated, and bacilli be later found in the urine which comes down through the catheter, and not in the urine which accumulates in the bladder, the diagnosis of monolateral renal tuberculosis may be regarded as established. That is to say, tuberculous disease in the opposite kidney may be excluded with approximate certainty. If bacilli appear in the catheter urine, and also in the bladder urine, under the circumstances mentioned, it is not conclusive proof of bilateral disease; for some urine from the tuberculous kidney may have passed the catheter and entered the bladder through the ureter proper. It seems wiser to take the chance of being thus placed in a dilemma, and having to catheterize the other ureter to discover if both kidneys are tuberculous, than to incur the comparatively useless risk of infecting a healthy ureter and kidney.

I purposely say nothing here as to the technique of catheterizing the female ureters, except to remark that with a patient properly placed in a modified dorsal position and under anesthesia, almost any one could pass the catheter with a little patience and practice, and with little difficulty were it not for the continual escape of urine into the bladder obscuring the field of observation—that is if the bladder lining be not markedly abnormal for some reason. Without anesthesia the difficulty mentioned can be largely obviated by placing the patient in the knee-chest position, but not every woman will submit, without anesthesia, to the manipulations necessarily incident to catheterization of the ureters. Dr. Kelly has an endoscope with an oblique, instead of a circular, orifice at the distal end which, with the patient in the genu-pectoral posture, can be fitted with some accuracy over the ureteral orifices and the urine collected separately with at least a fair degree of precision.

*Treatment.*—Of treatment I shall say little. The disease is so prone to become bilateral that an early surgical attack upon a single tuberculous kidney is almost always advisable. Nephrectomy, or nephrectomy with partial ureterectomy, or nephro-ureterectomy, is indicated according to the extent of the disease. It would seem, however, that conservatism, at least to the extent of preserving a doubtful portion of the bladder, is warranted and wise. Indeed it is by no means certain that tuberculous patches in bladders which have been infected from tuberculous kidneys may not recover under appropriate treatment, when the offending kidney has been removed.

Furthermore, genito-urinary tuberculosis is so frequently merely the local manifestation of a general tuberculosis, which, it may be, is elsewhere comparatively dormant, that the greatest caution should be exercised not to rush headlong into disappointing surgery. When the disease is known to be bilateral, no operation is of course advisable, unless it be undertaken for the relief of acute local manifestations on one side, such as a renal or perirenal abscess.

That cases get well, or at least remain quiescent, without surgical intervention is unquestionable. When for any good reason an operation is deemed inadvisable, the treatment recommended for tuberculosis elsewhere is by no means always without avail.

